# Supplementary material for: Are carbon emissions trading and green financial instruments synergistic? -Comprehensive quantitative research based on content analysis
Source: PLoS One. 2024 Mar 7;19(3):e0298601. doi: 10.1371/journal.pone.0298601 (PMC10919601; doi:10.1371/journal.pone.0298601)
Supplement: S1 Data — (ZIP) [file pone.0298601.s001.zip › Data/地级市Z_F工作报告.zip!/╔╜╢1⁄2/╘μ╫»╩╨/└┤╫╘╣1⁄2╓┌║┼í╛┬φ┐╦╩2╛▌═°í┐_2018.docx]

各位代表：

现在，我代表市人民政府向大会报告工作，请予审议，并请市政协各位委员提出意见。

一、2017年政府工作回顾

刚刚过去的2017年，面对宏观经济复杂形势、转型发展艰巨任务，全市上下认真学习贯彻党的十八大、十八届历次全会和十九大精神，深入践行习近平新时代中国特色社会主义思想，在市委的正确领导下，全力推进新旧动能转换重大工程，负重奋起、砥砺前行，取得了强市富民的新成就。预计全市生产总值完成2400亿元，增长6.7%；一般公共预算收入完成145.2亿元，增长1.2%，可用收入增长4%以上；固定资产投资完成1800亿元，增长5%；社会消费品零售总额达到980亿元，增长9.8%；城镇、农村居民人均可支配收入分别增长7.5%和8.6%。

（一）创新驱动形成加速度。协同创新取得“筑基架梁”式重大进展，启动了15平方公里鲁南产学研协同创新示范区规划建设；浙大山东工研院、北理工鲁南研究院正式运营，中科院化工新材料技术创新与产业化基地、常州大学国家级技术转移中心等一批创新平台加快落地；全市技术创新联盟、院士工作站、工程技术研究中心等达到165个。工业提质增效，编制实施12个重点产业五年发展规划，工业技改投资超过500亿元、增长15%以上；中材锂膜一期、丰元正极材料等项目建成投产，煤基新材料、人工智能、高端装备、锂电、光纤、医药和医疗器械等新兴产业加速膨胀，高新技术产业产值占比提高2.2个百分点；“智慧枣庄”建设迈出坚实步伐，鲁南大数据中心一期建成运营，互联网小镇入驻运营企业80家，山东省第四届互联网大会和数字中国（枣庄）峰会成功举办，区域性大数据基地正在隆起。服务业加快发展，现代物流、电子商务、共享经济等新业态蓬勃兴起，限上及规上服务业企业发展到1257家，现代服务业占服务业增加值的比重提升至45.3%；旅游综合收入增长14.2%。成功创建国家农业可持续发展试验示范区，市级以上新型农业经营主体达到917家，在全省率先基本实现农业现代化；打响“厚道枣庄人、放心农产品”品牌，滕州马铃薯入选全国百强农产品区域公用品牌，新增枣庄黑盖猪等“三品一标”66个。

（二）五场攻坚战赢得主动权。聚力打好社会稳定攻坚战，认真开展排查安全隐患防范四类风险活动和“四打击一整治”行动，扎实做好退役士兵安置，实现了“五个不发生”目标，为党的十九大胜利召开营造了安定和谐的社会环境。聚力打好安全生产攻坚战，全面推行三级巡查、异地执法，深入推进化工产业安全生产转型升级，集中整治超载超限，安全生产形势总体稳定。聚力打好生态环保攻坚战，办理完成中央环保督察转办件311件，依法关停整治“散乱污”企业325家；持续开展大气污染综合治理，“蓝繁”天数达到248天、较上年增加41天；全面落实河长制，关闭搬迁禁养区内畜禽养殖场979家；治理破损山体、塌陷地3.7万亩，完成造林8万亩。聚力打好脱贫攻坚战，落实市级专项扶贫资金1.1亿元，1.71万人实现脱贫。聚力打好金融风险防控攻坚战，妥善处置涉企金融隐患，守住了不发生系统性金融风险的底线。

（三）改革开放拓展活力源。市深改组先后召开8次会议，议定改革方案24个。深化供给侧结构性改革，压减煤炭产能64万吨，消化商业用房库存50万平方米；推动银企对接、煤电对接、大用户直供电扩面，高效使用接续还贷资金，累计为企业减负20多亿元；新申请马德里国际注册商标9件，新增山东名牌产品13个。加快农村改革步伐，农村产权交易体系建设通过国家验收，501个村完成集体产权改革试点任务。深化“放管服”改革，累计调整市级行政权力事项1782项、取消下放市级行政审批事项273项，市场主体发展到34.8万家、增加4.9万家。新引进保险、证券等金融机构5家，与恒丰银行签订战略合作协议；完成企业股改35家。务实举办北京、长三角等招商周活动，新开工外来固定资产投资亿元以上项目151个，其中10亿元以上项目10个。98个市级重点项目完成投资203亿元，万达广场、十电超超临界机组等31个项目建成运营。青岛港在我市设立内陆港区，预计全市进出口增长10%。开展第二届“枣庄英才”评选活动，新引进“千人计划”等高端人才17名。园区改革发展步伐加快，累计清理僵尸企业42家、盘活土地2400亩；枣庄高新区获批中国产学研合作创新示范基地，滕州经济开发区晋升国家级园区进入最后审批阶段。

（四）城乡建设呈现新气象。“多规合一”试点稳步推进，城市、乡镇总体规划编制完成。户籍人口城镇化率提高2.4个百分点。城市基础设施投资增长8.2%，建成海绵城市10.2平方公里，建设地下综合管廊6.1公里，完成城市建成区7条黑臭水体治理；新城体育中心投入使用，碧水绕城、南部路网工程启动建设。开工棚改安置房2.8万套，改造老旧小区108个。7个镇列入国家和省特色小镇、重点示范镇创建名单。累计建成美丽乡村872个，新建农村“户户通”道路2690公里，改造农村旱厕14万户。实施农村饮水安全巩固提升工程，惠及18.4万人。我市成为全省第二个实现镇街公交全覆盖的城市，入选全国公交都市创建城市。重大基础设施建设稳步实施，庄里水库完成投资24.4亿元，大坝主体工程和专项设施迁建有序推进；青檀路公铁立交桥竣工通车，十电运煤专线启动迁建；新台高速动工建设，枣菏高速、高铁换乘枢纽、快速公交B6线、枣木高速东延、沂台线南延等加快施工；枣庄机场完成规划选址，抽水蓄能电站纳入省选点规划，未来发展的支撑更加稳固。

（五）成果共享提升获得感。20件惠民实事有效落实，民生支出占比达到74.2%。新增城镇就业4.5万人，农村劳动力转移就业5.3万人。大力实施全民参保登记计划，基本实现法定人群全覆盖。农村低保、特困人员基本生活标准分别提高到每人每年3900元和5070元。化解教育“大班额”累计完成投资57.5亿元，开工校舍216.4万平方米，新增学位17.7万个。义务教育发展基本均衡县实现全覆盖。枣庄学院新校区开工，山东化工技师学院新校区启用。先后举办全市首届书香文化节、第二届群众文化艺术节，3件作品获省“文艺精品工程”奖；与山影集团合作，启动摄制电视剧《中兴》。创建成为省级文明城市，同步跨入全国文明城市提名城市行列。深入推进健康枣庄建设，公立医院综合改革稳步实施，药品加成全部取消，分级诊疗和医联体建设成效显著，北中医枣庄医院入选全国首批中医诊疗模式创新试点；成功举办市九运会、枣庄国际马拉松等体育赛事，参加第十三届全运会取得历史最好成绩。推进食安枣庄建设，食药安全监管体系逐步健全。实施“七五”普法，全民法治意识普遍增强。第三次农业普查圆满完成。双拥共建扎实推进。民族宗教、外事侨务、妇女儿童、老龄、残联、档案、史志、人防、地震、气象、红十字等事业均取得了新进展。

（六）从严治政筑牢防火墙。坚决贯彻全面从严治党新要求，严格履行“一岗双责”，认真执行新形势下党内政治生活若干准则和党内监督条例，管党治党政治责任有效落实。坚持民主集中制、“三会一课”等制度，扎实推进“两学一做”学习教育常态化、制度化。严格落实中央八项规定精神，驰而不息纠正“四风”，各级政府行政效能进一步提升。持续强化行政监察和审计监督，压减“三公”经费和一般性支出5%以上。监察体制改革有序展开。法治政府建设全面推进，《枣庄市山体保护条例》正式颁布。469件人大代表建议、政协提案全部办结。市长热线办复事项群众满意率达到97%，群众对政府工作的满意度持续提升。

各位代表！不忘初心，方得始终。近年来，面对资源型城市转型发展的现实困境，我们以海纳百川的开放胸襟，矢志不渝推进与名校、名企、名家的合作，深耕厚植产学研协同创新，探索完善人才引进、技术攻关、成果转化等体制机制，搭建平台、提供舞台、好戏连台，开启了资源型城市新旧动能转换的新征程。我们以咬定青山的战略定力，紧紧扭住发展第一要务，出台了加快工业创新发展、培育龙头骨干工业企业等一揽子政策，聚焦聚力打造“265产业集群”，心无旁骛抓招商、上项目、提质效，将实体经济向前推进了一大步，实现了老工业基地加速崛起的新突破。我们以事不避难的担当精神，成功争取了革命老区、淮河生态经济带、新一轮城市转型等扶持政策，实施了一批群众期盼的重大基础工程，解决了一些多年困扰发展的难题，结束了枣庄无国家级高新区、无A股上市企业、无直接通海港口的历史，增创了支撑枣庄长远发展的新优势。我们以枝叶关情的为民理念，每年办好一批事关群众就业、住房、入学、就医、养老的惠民实事，持续推动卫生城市、文明城市创建，坚决打好蓝天、碧水、青山保卫战，托起了群众安居乐业的新生活。

这些成绩的取得，得益于省委、省政府和市委的正确领导，得益于人大、政协和社会各界的监督支持，是全市人民团结一心、攻坚克难的结果。在此，我代表市人民政府，向奋斗在各行各业的广大干部群众，向解放军、武警驻枣官兵和中央、省驻枣单位，向在我市创业发展的投资者、建设者，向所有关心、支持枣庄发展的港澳台同胞和海内外朋友，表示衷心的感谢和崇高的敬意！

我们也清醒地认识到，当前我市发展不平衡不充分的问题还比较突出：传统产业转型升级亟待突破，新兴产业尚未形成强力支撑，距离打造一批千亿级、500亿级产业集群的目标尚有不小差距。企业整体创新能力不强，高附加值产品、名牌产品比重较低，高端人才数量不足，新旧动能转换任务艰巨。县域经济实力偏弱、发展不均衡，大项目、好项目偏少。经济外向度较低，园区发展活力不强。民生领域还存在薄弱环节，大气和水污染防治、生态修复任务繁重，脱贫攻坚、安全生产、社会治理压力较大，基本公共服务均等化水平还需进一步提升。全面从严治政仍需加强，作风建设面临新的考验，少数干部的担当精神、执行力度亟待提高等等。对于这些问题，我们将拿出更加精准有力的举措，认真加以解决，以新的发展成效回应全市人民的期盼和重托。

二、2018年政府工作任务目标

2018年是贯彻党的十九大精神的开局之年，是改革开放40周年，是决胜全面建成小康社会、实施“十三五”规划承上启下的关键一年。党的十九大描绘了我国未来发展的宏伟蓝图，全面规划了到本世纪中叶建成富强、民主、文明、和谐、美丽社会主义现代化强国的时间表和路线图，吹响了夺取新时代中国特色社会主义伟大胜利的前进号角。省第十一次党代会围绕“由大到强、走在前列”作出了一系列战略部署。今天的枣庄，正处在时代变革的关键节点，机遇当前，不容丝毫懈怠；重任在肩，更需扬帆奋进。

今年政府工作的总体要求是：全面贯彻落实党的十九大精神，以习近平新时代中国特色社会主义思想为指导，坚持稳中求进工作总基调，坚持新发展理念，紧扣社会主要矛盾变化，按照高质量发展的要求，以供给侧结构性改革为主线，深入实施新旧动能转换重大工程，激活创新、改革、开放三大引擎，推动质量、效率、动力三大变革，聚焦产业升级、乡村振兴、城市提质、生态改善、民生保障五大重点，锐意进取，埋头苦干，加快资源型城市创新转型持续发展步伐，全力建设自然生态宜居宜业新枣庄。

今年经济社会发展的主要预期目标是：全市生产总值增长7%左右；一般公共预算收入增长3%左右；外贸进出口稳定增长；城镇、农村居民人均可支配收入分别增长7.5%和8.5%左右；城镇登记失业率控制在3.5%以内；全面完成省下达的年度节能减排降碳约束性目标。

做好今年的政府工作，要把握好以下几点：一是凝聚共识、坚定信心，不议论。面对滚石上山、爬坡过坎的转型关口，我们要多一些脚踏实地、少一些品头论足，多一些反躬自问、少一些怨天尤人，多一些加油鼓劲、少一些妄自菲薄，像石榴籽一样紧紧抱团，打起精神、把正方向、加足油门，推动枣庄发展驶向跨越追赶的快车道。二是解放思想、大胆创新，不守旧。勇于冲破思想孤岛，以开放、包容、自强的心态看枣庄，以守正出新、自我革新、变中求新的行动自觉，摒弃单纯依靠资源和生产要素大规模高强度投入的增长方式，通过观念转变、要素转活、产业转新、企业转制、环境转优、全面转型，加速推动高速增长向高质量发展迈进。三是扭住目标、持之以恒，不懈怠。行百里者半九十。对看准了、定下来的事情，坚持一茬接着一茬干，既不能动辄改弦易调，也不能“夜晚想好千条路、天明照样卖豆腐”，必须起而行之、行则必至，以愚公移山的执着、水滴石穿的韧劲，坚定走好资源型城市创新转型持续发展的枣庄之路。四是担当负责、真抓实干，不推诿。不怕山高，就怕脚软。我们在各自的位置上，必须扛起肩上的担子，能挑千斤担、不挑九百九，迎着困难上、顶着风雨走，对有利于枣庄发展、有利于老百姓的事，只要坚决干、实打实地干、摽起膀子干、一刻不停歇地干，就一定能够抓实办成，就一定能够重塑枣庄的昔日辉煌。

三、加快新旧动能转换，全力推动经济高质量发展

深刻把握经济由高速增长向高质量发展转变的阶段性特征，坚持以“四新”促“四化”，改造提升传统动能，加快壮大新动能，构建实体经济、科技创新、现代金融、人力资源协同发展的产业体系，全面增强我市经济的创新力和竞争力。

（一）激活三大引擎，培育新型动力系统

一是在协同创新上求突破。牢牢抓住创新这个引领发展的第一动力，立足产业链布局创新链，着力打造以企业为主体、以市场为导向、“政产学研金服用”深度融合的创新体系。围绕创建国家可持续发展议程创新示范区，探索完善体制机制，破解绿色发展、可持续发展难题，提供可复制、可推广的“枣庄方案”。围绕建设鲁南产学研协同创新示范区，高水平编制规划，前瞻性布局功能，集聚众智、集成要素打造新旧动能转换的试验田、桥头堡。深入开展“创新平台建设年”活动，统筹推进浙大山东工研院、中科院化工新材料技术创新与产业化基地、北航机床创新研究院、北理工鲁南研究院、常州大学国家级技术转移中心、“千人计划”高新技术产业研究院、孵化器2.0科技创新产业公园、启迪之星孵化基地等高层次创新平台建设，加强与企业、项目对接，以“激水漂石”之势开辟蓬勃创新之局。强化企业创新主体地位，大力开展科技“小巨人”企业培育计划。年内，新增各类科技创新平台30个，科技型中小企业达到300家。

二是在改革任务落地上求突破。深化供给侧结构性改革。坚持破立结合推进“三去一降一补”，继续淘汰落后产能，大力破除无效供给；全面落实清费降税政策，推动大用户直供电扩面，提高接续还贷资金使用效益，进一步降低实体经济用能、融资、物流等成本。深化“放管服”改革。深入开展削权减证、流程再造、精准监管、体制创新、规范用权“五大行动”，打造审批事项少、办事效率高、服务质量优的营商环境。积极推进“最多跑一次”改革，实行“一窗受理、一网通办”，让群众少跑腿，让数据多跑路，真正“跑”出政府办事效率的加速度。全面实施市场准入负面清单制度，支持民营企业轻装上阵，促进非公有制经济健康发展。深化国企国资改革。坚持一企一策，通过引入战略投资者、整体上市、员工持股等方式，稳步推进混合所有制改革。整合煤电、建材、粮油、水务、交通运输等国有企业和资源，加快组建国有资本投资集团和产业集团。完成全市经营性国有资产统一监管和国有企业公司制改制。深化投融资改革。抓好财金控股、基础设施投资、鲁南（枣庄）经济开发投资等平台管理运营，增强滚动发展和资金供给能力。精心运作股权投资引导基金，扩大股权投资、债权投资规模。稳妥推进政府与社会资本合作，吸引更多社会资本参与公共服务领域项目建设。深化财税改革。建立市以下财政事权划分动态调整机制，实现“谁的财政事权、谁承担支出责任”。完善与财税贡献紧密挂钩的产业资金投向机制，壮大骨干财源、巩固特色财源、培植新兴财源。依法规范财政管理，防范政府债务风险。加快税收征管体制改革，推动国地税联合办税服务深度融合。

三是在打造开放型经济新优势上求突破。积极融入“一带一路”建设，坚持引进来和走出去并重，加快推进新一轮高水平对外开放。精准务实抓招商。围绕“265产业集群”培育，拉出重点产业、企业和项目招商清单；围绕国内外500强、行业100强、知名央企省企和大型民企，拉出企业投资意向清单；围绕京津冀、长三角、珠三角产业转移趋势，拉出产业承接清单，精准对接、登门招商，提高招商成功率。精心组织京沪、江浙、港台等境内外招商活动，积极筹备知名浙商枣庄行等经贸洽谈活动。全力支持兖矿、华电、中烟、中联等央企省企在枣庄新上项目、加快发展。完善招商引资政策，实行市级领导带队招商，建立健全跟踪考核、回溯奖励机制。多措并举促外贸。持续开展“双唤行动”，摸清底子、靠上服务，切实“唤回”更多出口业绩外流企业，“唤醒”更多零出口业绩企业。实施培育外贸骨干企业、培育自有出口品牌“双培工程”，提升出口质量和附加值。扶持外贸综合服务企业发展，大力开展跨境电商业务。推进外贸转型升级基地建设，巩固纺织服装、童车童床、橡胶轮胎等传统出口优势，扩大高端紧固件、数控机床等新兴出口份额。拓展青岛港枣庄内陆港区服务功能，启动海关特殊监管区建设，提高通关便利化水平。引导现有外资企业增资扩股，推动本土企业境外上市，提高利用外资水平。支持企业走出去开发资源、承包工程、开展加工贸易，更好地利用两个市场、两种资源。完善机制建项目。围绕省市级重点项目、央企省企合作项目，严格落实“一个项目、一名市级领导、一个责任单位、一抓到底”的推进机制，促进项目早日开花结果。积极争取省新旧动能转换基金，推动新旧动能转换重大工程取得突破性进展。

（二）实施四提工程，做优做强实体经济

一是传统产业提质效。坚持工业创新发展不动摇，通过科技嫁接改造、拉长产业链条，促进传统产业“老树发新芽”。加快打造千亿级产业集群。瞄准数控化、高端化、特色化，抓好北航航空发动机叶片磨削阵列机床、北理工精密微小型复合加工中心、腾龙不锈钢智能制造产业园、威达工业机器人关键部件生产线、亿和精密铸造等项目建设，打造高端装备制造产业基地，力争年内主营业务收入突破1000亿元。加快化工产业安全生产转型升级，推动鲁南高科技化工园区、薛城循环经济产业园通过首批省化工园区认定。着力推进鲁化120万吨甲醇、煤焦油馏分轻质化、盛隆化工搬迁改造等项目，做大化工基础原料规模；重点实施联泓新材料EVA扩产、潍焦薛城能源苯酐等项目，推动产业链条向高端新型化工领域延伸，创建国家级现代煤化工产业基地，力争“十三五”末成长为千亿产业板块。加快实施工业技改。深入开展工业企业技术改造三年行动计划，主动对标行业领先、企业最优，加速新技术、新工艺、新装备引进应用，全面提升煤电、轮胎、建材、造纸、纺织服装等传统产业。实施田陈富源热电联产、鲁化聚甲醛、精工电子二期等50个重点技改项目，年内工业技改投资增长15%以上。加快推进传统制造业智能化。促进“两化”深度融合，推动“企业上云”，积极培育个性化定制、共享制造等“互联网+制造业”新模式，打造一批新型工业化产业示范基地。支持企业加快数字化、网络化、智能化改造，建设无人生产线、智能工厂，推动“机器换人”，培树一批智能制造试点企业，加快制造业强市步伐。

二是新兴产业提规模。紧密对接“中国制造2025”，聚集高端创新人才、创业项目，推动新兴产业“雨后春笋”般节节拔高，打造一批百亿级产业基地。快速引爆大数据产业。将其作为全市重要发展战略，依托鲁南大数据中心开展产业招商，引进一批大数据存储、应用及服务企业，培育大数据核心业态，种下“智慧树”，开出“钻石矿”，打造区域性大数据产业基地。加快推动“16朵云”建设运营，打造“100个应用场景”，发展大数据关联业态。高标准建设运营互联网小镇，吸引创客团队聚集，打造京沪高铁经济带“数谷”。继续办好数字中国（枣庄）峰会和山东省互联网大会，开启大数据时代的“枣庄时间”。快速膨胀新材料产业。加快推进东方光源总投资30亿元的棒纤缆项目，建设北方地区最大的光纤基地。重点实施联润生态环保纱、青纺联功能性纤维等项目，打造纺织新材料产业基地。抓好北玻院科技成果转化基地建设，实施轨道交通复合材料、航天特种树脂等项目，带动新材料产业整体跃升。快速做大医药及医疗器械产业。支持三九、益康等企业扩大市场份额，加快海王医疗器械产业园、苏鲁海王医药物流中心、国家药典博物馆及健康产业园、汤姆科技微环境护理等项目建设，进一步壮大产业规模。快速做强锂电产业。抓好中材锂膜、华亿比科动力电池等项目建设，完善上下游配套，打造锂电产业基地。快速发展节能环保产业。抓好生物质发电、水处理剂等项目建设，培育环境检测、节能推广等中介服务企业，打造具有枣庄特色的节能环保产业集群。

三是跨界融合提潜能。推进服务业与制造业融合。大力发展工业设计、融资租赁等生产性服务业，促进产业相互渗透、创新提升。加快推进枣庄铁路物流园、高速物流配送服务园区规划建设，提升运河沿线港航物流产业园，促进物流、配送、制造协同发展。推进传统业态与互联网融合。大力发展平台经济、共享经济，推动真爱商城等专业市场“触网”发展。实施“电子商务进农村综合示范县”建设，培育更多的“淘宝村”，让农民“借网”致富。推进金融与实体经济融合。加快市普惠金融服务中心建设，积极创建国家级普惠金融改革试验区。深化地方法人金融机构改革。继续推动“银税互动”“政银保贷款”，鼓励金融机构创新信贷产品。年内引进股份制银行和保险、证券机构5家以上，构建多元化、多层次金融体系。推进医养融合。鼓励医疗机构和社会资本兴办健康养生产业，加快市医养康复中心、北中医医养结合园等项目建设，努力打造区域性医养高地。推进军民融合。围绕通用航空装备、数字信息、智能机器人、特种工程轮胎等重点领域，推动“军转民”“民参军”，促进军民融合产业集聚化、规模化发展。

四是品牌高端提价值。充分发扬“墨子发明”“鲁班创造”的“工匠精神”，瞄准价值链中高端，打造一批有特色、有价值、有底蕴的枣庄品牌和“金字招牌”。发挥国家锂电池、机床质量监督检验中心的引领作用，带动企业建立专业标准化体系和全面质量管理体系。主动对接山东品牌全球行、中华行、网上行系列活动，积极争创中国驰名商标、省长质量奖、山东名牌产品，力争商标有效注册总量增长10%以上、新申请马德里国际注册商标5件以上。加强商标、专利保护，打通知识产权创造、运用、保护、服务全链条，助力经济高质量发展。

（三）夯实四大支撑，筑牢转型发展根基

一是狠抓园区提升。加大市级统筹力度，聚集各类资源要素，增强园区载体功能。突出“双核”引领。支持枣庄高新区依托“一园一城两镇一谷一中心”，规划建设浙大科技产业园、中移铁通智能产品产业园等一批“区中园”，努力建成体制机制改革的先行区、创新创业要素的集聚区、新兴产业培育的核心区。支持滕州经济开发区完善“一区多园”模式，明确主导产业，抬高发展标杆，挖掘发展潜能，尽快跨入国家级经济开发区行列。突出多点并进。明确各省级开发区、专业特色园区定位，统筹产业布局和产业衔接，集中力量谋划实施一批关联度高、带动力强的核心产业项目。强化园区招商职能，完善利益分享机制，推动重大项目有序流动、飞地布局，做强县域经济“发动机”。突出机制创新。加快开发区财权、事权改革，推行职员制、聘任制，健全多元化投融资体系。加大僵尸企业清理力度，实现腾笼换鸟、凤凰涅槃。完善园区考核指标体系，实行排名通报制度，激励园区争先晋位、竞相发展。
